# Supplementary material for: Waterborne and Dietary Bioaccumulation of Organophosphate Esters in Zooplankton Daphnia magna
Source: Int J Environ Res Public Health. 2022 Jul 31;19(15):9382. doi: 10.3390/ijerph19159382 (PMC9367849; doi:10.3390/ijerph19159382)
Supplement: Supplementary file 1 [file ijerph-19-09382-s001.zip › ijerph-1829861-supplementary.pdf]

Supporting Information

Waterborne and dietary bioaccumulation of organophosphate esters in Zooplankton *Daphnia magna*

Wenxin Liu, Hong Zhang, Jiaqi Ding, Wanyu He, Lin Zhu * and Jianfeng Feng *

Key Laboratory of Pollution Process and Environmental Criteria of Ministry of Education and Tianjin Key Laboratory of Environmental Remediation and Pollution Control, College of Environmental Science and Engineering, Nankai University, Tianjin 300071, P. R. China

Corresponding author:
zhulin@nankai.edu.cn (L.Z.); fengjf@nankai.edu.cn (J.F.)

Contents

Number of Pages: 9

Page S2: Sample preparation

Page S3: Instrumental analysis

Page S4-S8: Table S1-Table S5

Page S9-S12: Figure S1-Figure S4

Sample pretreatment

In brief, the samples were spiked with 5 ng of d_{12}-TCEP, d_{27}-TNBP and d_{15}-TPHP as surrogates. They were soaked with 4 mL ACN for 3 h，and extracted twice using 4 mL, 3 mL of ACN continuously by ultrasonication for 20 min each time and centrifuged at 2000 g for 10 min. The supernatant was combined in another glass tube, dried under a nitrogen stream, and redissolved with 2 mL Hexane. The extract was passed through a GCB/NH2 cartridge (200 mg/200 mg, 6 mL, CNW, China) at a rate of 1-2 drop per second for cleanup. The cartridge was previously conditioned with 5 mL of methanol, 5 mL of DCM and 5mL of hexane. The cartridge was then eluted with 5 mL of Hexane/DCM 1:1 (v/v) and 5 mL of DCM. The collected solution was evaporated under a gentle stream of N2 for dryness, and transferred to 1 mL of ACN / Milli-Q water [9:1 (v/v)]. After passing through a 0.22μm filter membrane, the supernatant was taken out for UPLC-MS/MS analysis.

The exposure solution was spiked with 5 ng of d_{12}-TCEP, d_{27}-TNBP and d_{15}-TPHP as surrogates, and extracted by a solid phase extraction cartridge (ENVI-18, 500 mg 6 mL, Supelco, USA). Prior to extraction, the cartridge was preconditioned with 10 mL of DCM, 10 mL of ACN and 10 mL of Milli-Q water. The solution was loaded on the cartridge at approximately 1-2 drop per second. The cartridge was eluted with 10 mL of DCM / ACN mixture [1:4 (v/v)]. The eluent was blown to near dry under a gentle stream of nitrogen, and transferred to 1 mL of ACN / Milli-Q water [9:1 (v/v)]. After passing through a 0.22μm filter membrane, the supernatant was taken out for UPLC-MS/MS analysis [1, 2].

Instrumental analysis

An UPLC-MS/MS system (Xevo TQ-S; Waters, Milford, MA, USA) was used to quantify OPEs in the samples. Chromatographic separation of OPEs was accomplished on a Waters BEH C18 column (2.1 mm × 50 mm, 1.7 μm) coupled with a VanGuard Pre-column (C18 column, 2.1 mm × 5 mm, 1.7 μm). The injection volume was 10 μL, and the column temperature was 55 °C. For the gradient elution, a binary eluent of water (A) and acetonitrile (B), both containing 0.1% formic acid, was used for the separation of analytes at a flow rate of 0.4 mL/min. The gradient was set as follows (with reference to B): 0 min 10% B, 1-3.5 min 50% B, 3.6 min 40% B, 4.1-6 min 50% B, 7-9 min 100% B and 10 min 10% B.

Chromatograms were recorded using positive ion mode and multiple reaction monitoring. Nitrogen was applied as desolvation gas and argon as collision gas. Other operation parameters for MS were set as follows: capillary voltage 3.5 kV; source temperature 150 °C; probe temperature 400 °C; cone gas flow 150 L/h; and desolvation gas flow 800 L/h. The detection parameters of each pollutant are listed in Table S1 [1, 2].

Table S1. Specific UPLC-ESI$^+$-MS/MS parameters of OPEs for MRM detection.

Compounds	Retention time(min)	Monitoring transition	Internal standard	Cone voltage (V)	Collision energy(V)
TCEP	1.50	285>155	TCEP-d12	10	18
		285>223*		10	12
TDCPP	2.96	429>99	TCEP-d12	14	20
		429>209*		14	12
TPHP	3.35	327>77	TPHP-d15	8	34
		327>152*		8	32
TBOEP	5.29	399>199*	TNBP-d27	12	12
		399>299		12	10
**Internal Standards**					
TCEP-d12	1.49	297>102*		10	18
TPHP-d15	3.25	342>160*		8	32
TNBP-d27	3.47	249>102*		18	16

*: Quantification ion.

Table S2. Recoveries of OPEs.

	TCEP	TDCPP	TBEOP	TPHP
Daphnia magna (%)	92.88 ± 10.39	90.46 ± 5.42	113.34 ± 3.02	107.19 ± 12.94
Scenedesmus obliquus (%)	85.31 ± 9.25	77.46 ± 4.55	106.27 ± 0.90	73.40 ± 2.06
Media (%)	95.67 ± 20.43	103.65 ± 4.78	109.01 ± 13.60	91.16 ± 4.37

Table S3. The method quantification limits (MQLs).

	TCEP	TDCPP	TBOEP	TPHP
Daphnia magna (ng/g)	182.61	86.96	47.83	34.78
Scenedesmus obliquus (ng/g)	177.78	155.56	91.11	37.78
Media (ng/L)	67.93	121.86	68.53	120.35

Table S4. The actual concentration in waterborne exposure experiment (μg/L).

	TCEP	TDCPP	TBOEP	TPHP
Low group (20 μg/L)	25.27±0.97	16.11±1.26	16.71±2.54	15.71±0.41
High group (100 μg/L)	113.63±0.86	119.87±3.37	136.35±2.07	98.56±1.59

Table S5. Chemical and physical properties of OPEs [3].

	TCEP	TDCPP	TBOEP	TPHP
Solubility in water (mg/L) at 25 ºC	7000	7	1100	1.9
Vapor pressure (mmHG) at 25 ºC	0.0613	2.98×10^{-7}	2.13×10^{-6}	1.12×10^{-5}
Henry's law constant (atm m3 mol^{-1}) at 25 ºC	1.673×10^{-7}	1.080×10^{-7}	3.285×10^{-7}	1.960×10^{-7}
Log K_{ow}	1.63	3.65	3.00	4.70

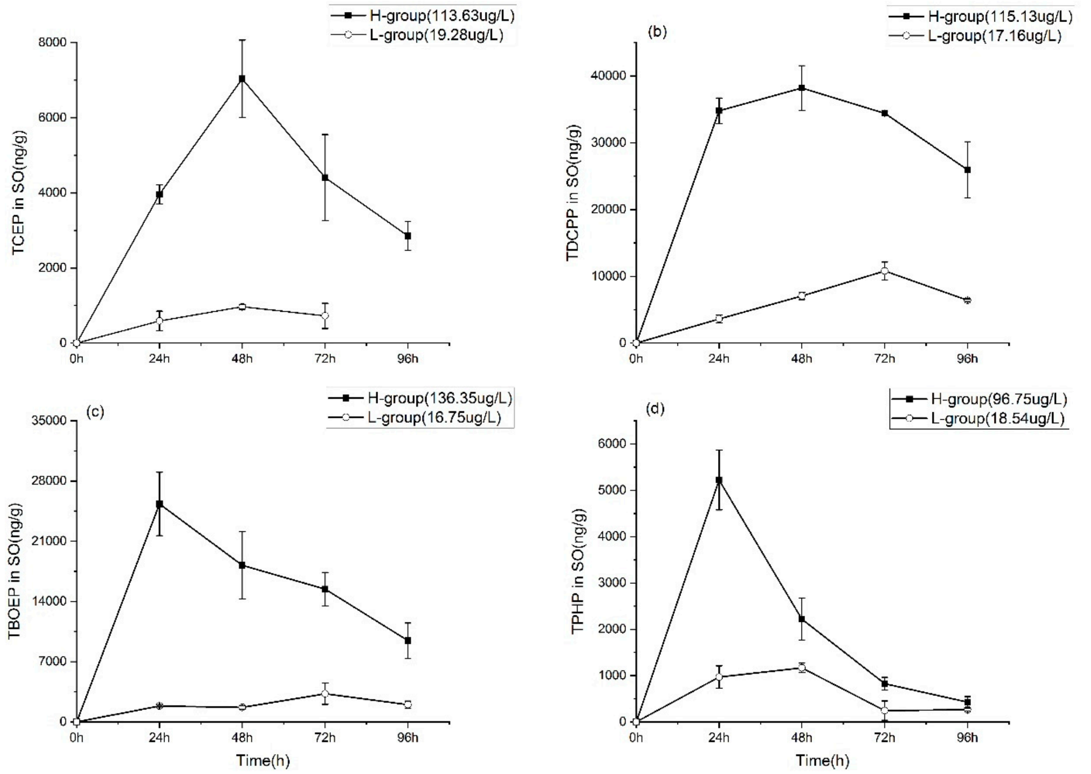

Figure S1. OPEs ((a) TCEP, (b) TDCPP, (c) TBOEP, (d) TPHP) in *Scenedesmus obliquus* (SO) (ng/g dw). Solid points represent the high concentration group (100μg/L) and hollow points represent the low concentration group (20μg/L). The actual exposure concentration has been shown in the legend. The values were the means ± SD of three replicates.

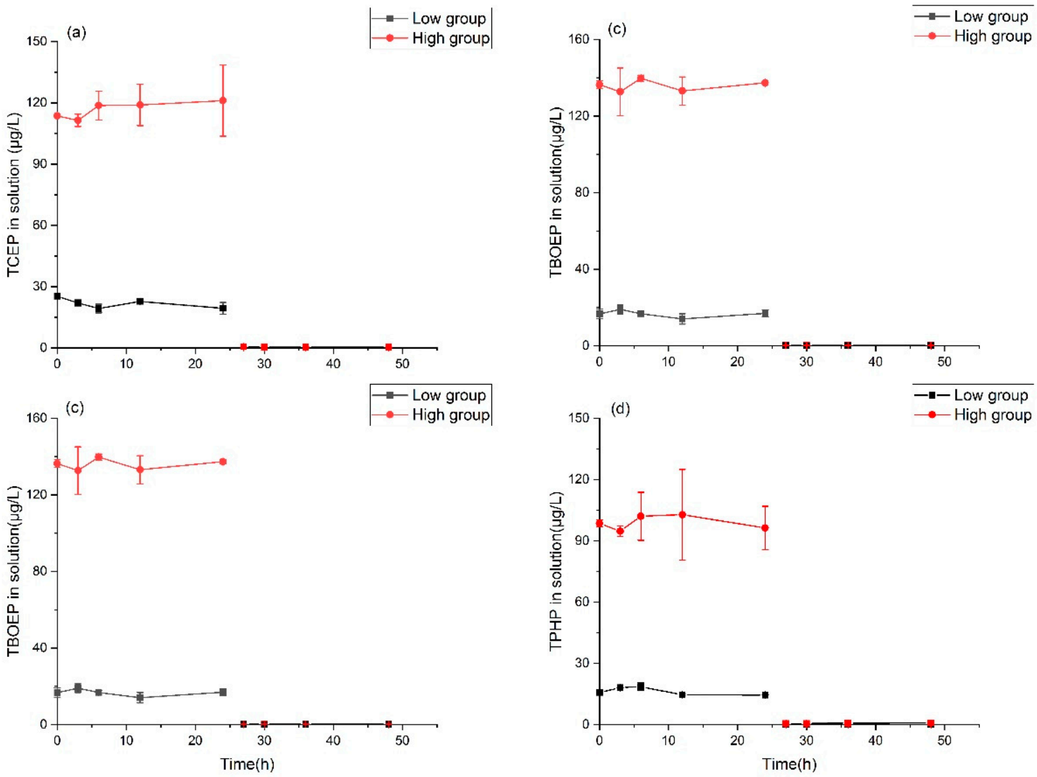

Figure S2. OPEs ((a) TCEP, (b) TDCPP, (c) TBOEP, (d) TPHP) in exposure solution during the waterborne exposure experiment (μg/L). Black lines and points represent the low concentration group (20μg/L) and red lines and points represent the high concentration group (20μg/L). The values were the means ± SD of three replicates.

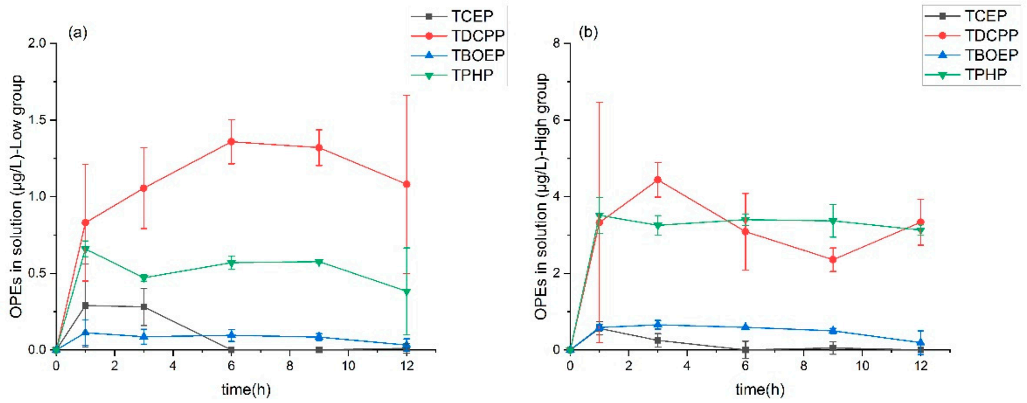

Figure S3. Concentration of OPEs in the solution during the dietary experiment (μg/L). (a) OPEs in solution at low exposure concentration (20μg/L). (b) OPEs in solution at high exposure concentration (100μg/L). The values were the means ± SD of three replicates.

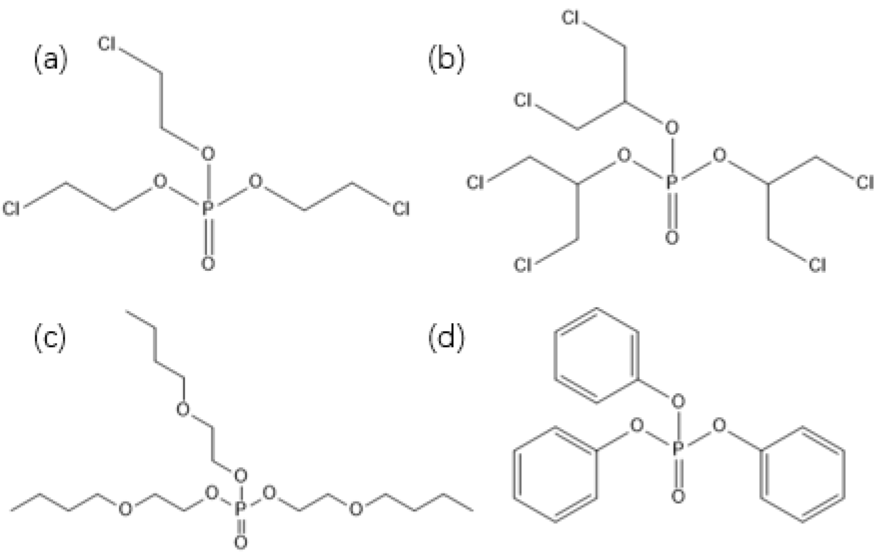

Figure S4. Structure of four OPEs ((a) TCEP, (b) TDCPP, (c) TBOEP, (d) TPHP).

Reference

1. Liu, Q.; Wang, X.; Yang, R.; Yang, L.; Sun, B.; Zhu, L., Uptake Kinetics, Accumulation, and Long-Distance Transport of Organophosphate Esters in Plants: Impacts of Chemical and Plant Properties. *Environ Sci Technol* **2019,** 53, (9), 4940-4947.
2. Wang, X.; Zhong, W.; Xiao, B.; Liu, Q.; Yang, L.; Covaci, A.; Zhu, L., Bioavailability and biomagnification of organophosphate esters in the food web of Taihu Lake, China: Impacts of chemical properties and metabolism. *Environ Int* **2019,** 125, 25-32.
3. Hou, R.; Xu, Y.; Wang, Z., Review of OPFRs in animals and humans: Absorption, bioaccumulation, metabolism, and internal exposure research. *Chemosphere* **2016,** 153, 78-90.
